# Engineering antibody-like inhibitors to prevent and treat HIV-1 infection

**DOI:** 10.1097/COH.0000000000000367

**Published:** 2017-02-21

**Authors:** Matthew R. Gardner, Michael Farzan

**Affiliations:** Department of Immunology and Microbiology, The Scripps Research Institute, Jupiter, Florida, USA

**Keywords:** adeno-associated virus gene therapy, alternative HIV vaccine, bispecific antibody, eCD4-Ig

## Abstract

**Purpose of review:**

Here we discuss recently developed HIV-1 entry inhibitors that can target multiple epitopes on the HIV-1 envelope glycoprotein (Env), with an emphasis on eCD4-Ig. Some of these inhibitors are more potent and broader than any single antibody characterized to date. We also discuss the use of recombinant adeno-associated virus (rAAV) vectors as a platform for long-term expression of these inhibitors.

**Recent findings:**

Much of the exterior of HIV-1 Env can be targeted by broadly neutralizing antibodies (bNAbs). Recent studies combine the variable regions or Fabs from different bNAbs, often with the receptor-mimetic components, to create broad, potent, and hard-to-escape inhibitors. rAAV vectors can express these inhibitors for years *in vivo*, highlighting their ability to prevent or treat HIV-1 infection.

**Summary:**

By targeting multiple epitopes on Env, bispecific and antibody-like inhibitors can be broader and more potent than bNAbs. These inhibitors can provide long-term protection from, and perhaps suppression of, HIV-1 if they are administered by a delivery platform, like rAAV vectors, but only after rAAV limitations are addressed.

## INTRODUCTION

The HIV-1 envelope glycoprotein (Env) is a trimer of gp120/gp41 heterodimers that mediates viral entry. Env binds cellular CD4 and a tyrosine-sulfated coreceptor, primarily CCR5 or CXCR4, to translocate the viral capsid core across the host cell membrane (reviewed in [1]). As the sole viral protein expressed on the viral membrane, Env has been the main target of HIV-1 vaccine design. Although a conventional HIV-1 vaccine remains elusive, our understanding of broadly neutralizing antibodies (bNAbs) has significantly transformed immunogen design. Epitope mapping of bNAbs on Env has also shown that many distinct functional epitopes are exposed on Env (reviewed in [2]). The broadest and most potent bNAbs target the CD4-binding site, the base of the V3 loop (variable loop 3) around the N332 glycan, the apex of the V2 loop (variable loop 2), the membrane proximal external region (MPER), or the gp120/gp41 interface. Among these are the CD4-binding site antibodies VRC01 and 3BNC117 and the V3 glycan antibodies 10-1074 and PGT121. Individually, these bNAbs have clear therapeutic efficacy in rhesus macaques [3^▪^,^4^^▪▪^,^5^–^7^]. Furthermore, VRC01, 3BNC117, and 10-1074 have already shown promise in human clinical trials [8,9^▪^,10^▪^,^11^–^13^].

**Box 1:**
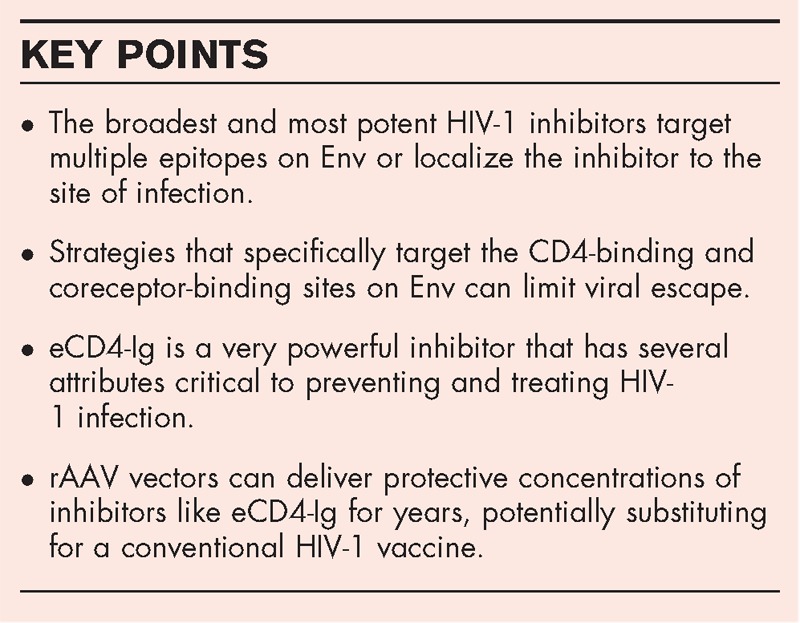
no caption available

## DESIGNING INHIBITORS THAT TARGET MULTIPLE ENV EPITOPES

Despite the numerous successes with bNAbs, there are obvious flaws with the use of single bNAb. No bNAb can neutralize all HIV-1 isolates, and most of the best characterized bNAbs fail to neutralize more than 30% of isolates tested with 80% inhibitory concentrations (IC_80_s) > 20 μg/ml. The two broadest antibodies, N6 and 10E8, cover 98% of isolates assayed with 50% inhibitory concentrations (IC_50_s) < 50 μg/ml [14^▪▪^,^15^]. Even these exceptional cases underscore the fact that many isolates have already escaped these antibodies. Thus, treatment with a single antibody will rapidly select for mutations that render the antibody ineffective for several reasons. First is the high rate of mutation mediated by HIV-1 reverse transcriptase. Second, every bNAb has contact residues within their epitopes that are not necessary for Env function and not conserved. Third, because every Env has escaped similar antibodies in its recent past, there are easily accessible escape pathways requiring minimal mutations. Although less acute, escape remains a concern for prevention should resistant viruses become more frequent in an antibody-treated population. To overcome the weaknesses of individual bNAbs, studies have sought to optimize bNAb cocktails. Most cocktails combine antibodies that target distinct epitopes to increase breadth or that work synergistically to increase potency [16,17^▪^,^18^–^20^]. In a recent study, Wagh *et al.* [16] determined the best triple and quadruple antibody combinations against a panel of 200 clade C isolates. These types of studies suggest that at least three bNAbs will be necessary for preventing and treating HIV-1 infection.

**FIGURE 1:**
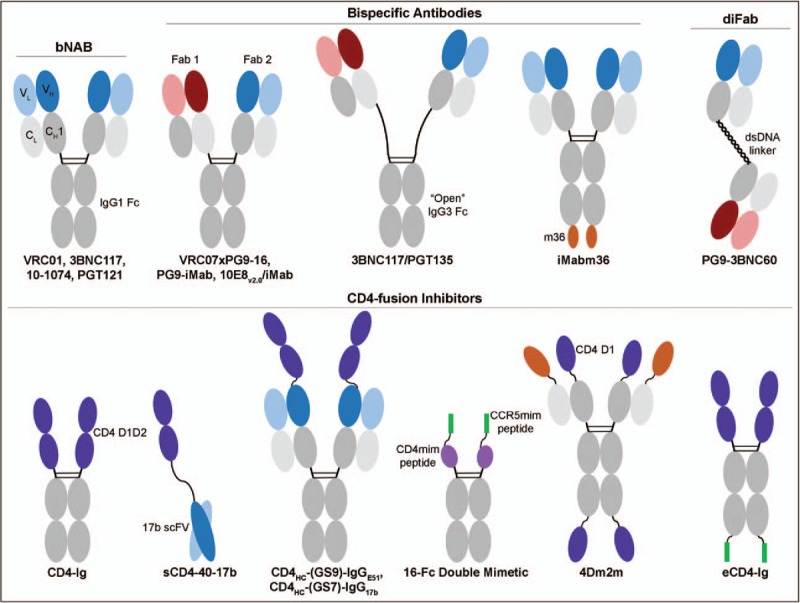
Designs of HIV-1 inhibitors that target multiple epitopes on HIV-1. Broadly neutralizing antibodies, bispecific antibodies, diFabs, and CD4-fusion inhibitors are represented, with examples named below each basic design. bNAb, broadly neutralizing antibody; CCR5mim, CCR5-mimetic sulfopeptide (green); CD4 D1D2, CD4 domains 1 and 2 (dark purple); CD4mim, CD4-mimetic (light purple); C_H_1, constant heavy chain 1 (gray); C_L_, constant light chain (light gray); dsDNA, double-stranded DNA; Fab, fragment antigen-binding; Fc, fragment crystallizable region (gray); IgG1, immunoglobulin 1; IgG3, immunoglobulin 3; scFv, single-chain variable fragment; VH, variable heavy chain (blue or red); VL, variable light chain (light blue or light red).

Although targeting different epitopes on Env with multiple bNAbs is an effective strategy, efforts have been made to design single inhibitors that simultaneously target multiple sites on Env, including bispecific antibodies that combine two different bNAbs in a single construct (Fig. 1). For example, bispecific antibodies have been generated that combine VRC07 (CD4-binding site) with PGT121 (V3 glycan), 10E8 (MPER), or PG9-16 (V2 apex) and 10E8 with PG9-16 [21]. Recently, Bournazos *et al.* [22^▪^] demonstrated the utility of the IgG3 Fc for creating bispecific antibodies. To accommodate the distance between two bNAb epitopes on Env, all the cysteines in the IgG3 hinge except two were mutated to serines. The authors concluded that the open IgG3 design could be used to increase the potency and breadth of other bNAbs. A separate study by Galimidi *et al.* [23] also showed that spacer length was crucial for a single inhibitor to target two epitopes. In this study, two Fabs were linked by dsDNA to form diFabs. Among the different diFabs generated, the hetero-diFab of PG16-3BNC60 with a 50-bp linker that targeted the CD4-binding site and V2 apex exhibited the greatest synergy. Together, these studies show that linkers of appropriate length are key to developing inhibitors that target two Env epitopes.

Although these traditional bispecific antibodies target two epitopes on Env, some of the most potent bispecific antibodies have taken a more unusual approach. The laboratory of David Ho has developed bispecific antibodies that target Env with one arm and an HIV-1 receptor or coreceptor with the other arm. Initially, single chain variable fragments (scFv) of PG9 or PG16 were combined with the heavy-chain N-terminus of ibalizumab (iMab), a humanized antibody that targets domain 2 of cellular CD4 [24]. Both bispecific antibodies, PG9-iMab and PG16-iMab, neutralized an entire panel of 118 HIV-1 isolates. Interestingly, these bispecific antibodies also neutralized HIV-1 isolates that were resistant to the component iMab, PG9, or PG16 antibodies. In a more recent study, Huang *et al.* [25^▪▪^] expanded their bispecific repertoire by generating numerous antibodies that combined one arm from a bNAb with either iMab or Pro140 (P140), an antibody that targets the coreceptor CCR5. Their most potent inhibitors combined the MPER antibody 10E8 with iMab (10E8/iMab) or P140 (10E8/P140) that had mean IC_50_ values of 2.0 and 1.0 ng/ml, respectively, when assayed against 118 HIV-1 isolates. 10E8/iMab exhibited 100% breadth with this panel, whereas 10E8/P140 had 99% coverage. These two studies demonstrate the effectiveness of localizing the Env-binding inhibitor directly to the site of infection.

## INHIBITORS THAT TARGET THE CONSERVED CD4-BINDING AND CORECEPTOR-BINDING SITES

It remains unclear how effective bispecific antibodies will be if the virus can already escape from both arms of the antibody. Indeed, these antibodies are at best equivalent to bNAbs in patients harboring resistance to one arm. Consequently, other groups have sought to target two of the most conserved sites on Env, the CD4-binding, and coreceptor-binding sites [26–31]. The conservation and functional roles of these sites make them ideal targets for inhibitor designs.

Soluble CD4 (sCD4) and its more bioavailable form, CD4-Ig, comprises the first two domains of CD4 (Fig. 1). In the case of CD4-Ig, these domains are fused to an IgG Fc. In addition, smaller CD4-like domains, based on CD4 domain 1 [32] or selected from a scorpion toxin scaffold [33], have been developed. To target the coreceptor-binding site, investigators have used constructs derived from CD4-induced (CD4i) antibodies, including 17b [34], E51 [35,36], and m36 [37], whose epitopes are more exposed in the presence of CD4 or sCD4. Some CD4i antibodies, such as E51, share with CCR5 a series of sulfated tyrosines critical to their ability to bind Env [36]. The first inhibitors to target the receptor-binding sites fused sCD4 to the scFv of 17b (sCD4-40-17b) [38]. Similarly, West *et al.* [39] created sCD4 fusion constructs by fusing sCD4 to the N-terminus of E51 or 17b heavy chains of a full-length antibody. Two of these, CD4_HC_-(GS9)-IgG_E51_ and CD4_HC_-(GS7)-IgG_17b_, were shown to be more potent than CD4-Ig. The importance of linker length was further highlighted by Quinlan *et al.* [40] who described a double-mimetic inhibitor that linked a sulfated CCR5-mimetic peptide and a CD4-mimetic peptide fused to an IgG1 Fc. The double mimetic peptide (16-Fc) derived its potency from simultaneously binding both the CD4-binding and coreceptor-binding sites on monomeric gp120. Recently, Chen *et al.* [41] characterized a potent bispecific fusion protein – 4Dm2m – that combined elements of a stable CD4 domain 1 (D1.22) with the antibody domain of m36. Sun *et al.* [42] took a different approach by fusing m36 to the C-terminus of iMab (iMabm36), thus creating an inhibitor that targeted the CD4i epitope and localized to CD4^+^ cells.

A key weakness of most of the inhibitors described is that one or both of their domains bind epitopes that contact variable Env residues. These unconserved contact residues serve as easy targets for viral escape. In contrast, our lab has developed eCD4-Ig [43^▪▪^], which is CD4-Ig with a short tyrosine-sulfated CCR5-mimetic peptide [44,45] appended to its C-terminus. Its Env binding sites are thus small, conserved receptor-binding regions. eCD4-Ig is broader than any HIV-1 antibody described, potently neutralizing all 73 HIV-1 isolates initially tested, regardless of clade or coreceptor preference, as well as all SIV and HIV-2 isolates assayed. These included many isolates resistant to CD4-Ig and to the CD4-binding site antibodies VRC01, 3BNC117, and NIH45–46. We have since shown that eCD4-Ig also neutralizes an entire 200 isolate clade C panel. Interestingly, eCD4-Ig utilizes both of its CCR5-mimetic sulfopeptides as well as one CD4 arm to bind Env and neutralize HIV-1 isolates. In addition to engaging Env, these sulfopeptides prevent CD4-Ig from enhancing infection at low concentrations or when cellular CD4 was limiting. Moreover, eCD4-Ig is harder to escape than antibodies. After more than 60 passages *in vitro* under conditions that readily elicited escape from NIH45–46 and CD4-Ig, we observed partial resistance but no escape from eCD4-Ig. Remarkably, the eCD4-Ig-selected swarm was fully resistant to CD4-Ig. The difficulty of escape and the inability to identify resistant isolates highlight the two key features of eCD4-Ig. First, HIV-1 has never encountered an inhibitor like eCD4-Ig and does not appear to have an accessible pathway for escape. Second, there appears to be a clear fitness cost for escape eCD4-Ig, likely because of the close similarity between eCD4-Ig and the native receptors of HIV-1.

Another advantage of eCD4-Ig is the size of its gene (∼1.38 kb). This feature has allowed eCD4-Ig to be combined with viral vectors with tight limitations on transgene size, specifically recombinant adeno-associated virus (rAAV). We delivered a rhesus macaque version of eCD4-Ig (rh-eCD4-Ig) using rAAV vectors to four rhesus macaques [43^▪▪^]. The macaques expressed rh-eCD4-Ig for almost a year at 17–77 μg/ml. These levels of rh-eCD4-Ig were able to protect all four macaques from six escalating SHIV-AD8 challenges that infected all four control macaques, up to 16 times the 50% animal infectious dose of this virus. In a follow-up study, we demonstrated that low levels of rh-eCD4-Ig protected four eCD4-Ig-inoculated animals from challenge doses of SIVmac239 that infected all eight control macaques. These studies show that a one-time inoculation with AAV-eCD4-Ig can protect from high doses of divergent, neutralization resistant viruses for at least 1 year after inoculation. Studies of rAAV with other proteins suggest that protective concentrations could last for 5 years or more. Thus, although work on conventional vaccines remains slow, effective, universal, and long-term protection from HIV-1 may be more quickly accessible with rAAV and eCD4-Ig.

## OVERCOMING THE HURDLES OF RECOMBINANT ADENO-ASSOCIATED VIRUS VECTORS

Despite the potential of AAV-eCD4-Ig, there are concerns with rAAV vectors that must be addressed before human trials can be initiated. rAAV vectors have been examined for safety in numerous clinical trials and are currently being used to treat hemophilia [46,47]. These vectors do not replicate or integrate and are generally considered well tolerated. However, their small gene cassette size (about 5.0 kb) limits their applications. Full-length antibodies can fit into a single-stranded rAAV vector, either using two promoters or with an F2A peptide separating the heavy and light chains [48–50]. However, bispecific antibodies require two heavy-chain and light-chain arms to bind different epitopes. Thus, the use of bispecific antibodies with rAAV vectors would require at least two different vectors. eCD4-Ig itself easily fits into rAAV vectors. However, we have observed that TPST2, the enzyme necessary for sulfating the CCR5-mimetic peptides, is necessary for eCD4-Ig's full activity *in vivo*. Fortunately, TPST2 is only 1.14 bp, thus a single rAAV can accommodate both *eCD4-Ig* and *TPST2* genes. Although our first *in vivo* studies provided eCD4-Ig and TPST2 in different vectors, we are currently evaluating rAAV vectors that contain both genes.

**FIGURE 2:**
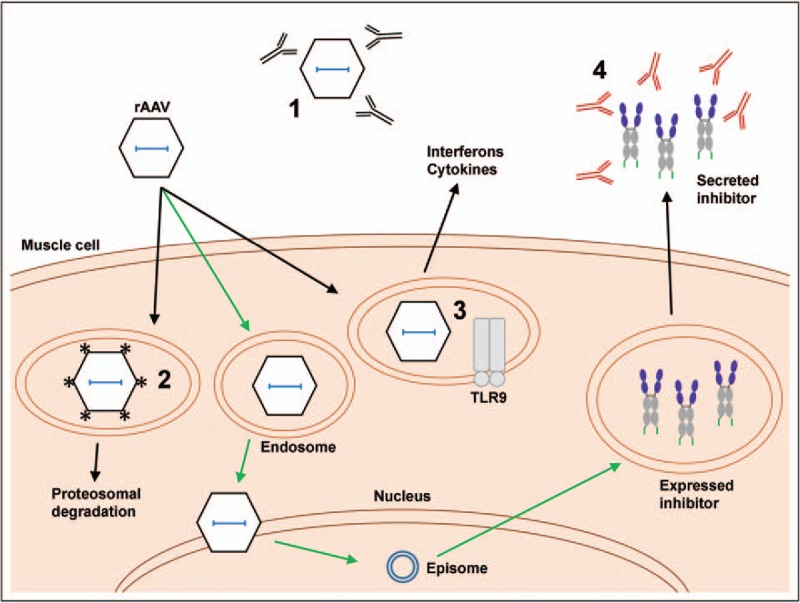
Summary of the current challenges for rAAV vector delivery. Preexisting immunity against AAV capsids will result in rAAV vectors that are neutralized before transducing the muscle cell. Upon entry into the muscle, rAAV vectors can get shuttled to the nucleus. However, rAAV capsids can be phosphorylated and targeted for premature degradation, or the TLR9 pathway can be activated leading to the production of interferons and proinflammatory cytokines. The rAAV vectors that reach the nucleus release their genomic material that forms episomes. The episome expresses the delivered inhibitor transgene that is secreted by muscle. The secreted inhibitors can also be targeted by host antibodies, limiting inhibitor levels in circulation, or by cytotoxic T cells, eliminating expressing cells entirely. Green arrows indicate pathway for production of inhibitor; black arrows indicate pathways that activate the immune response to limit transduction or inhibitor expression. rAAV, recombinant adeno-associated virus; TLR9, Toll-like receptor 9.

There is also the concern that the host immune response will limit transduction efficiency from rAAV vectors (Fig. 2) [51–54]. For example, there is high preexisting immunity to typical rAAV vectors that use AAV1 (67%), AAV2 (72%), and AAV8 (38%) capsids [55]. Multiple groups are working on creating new AAV capsids for greater transduction either by rational design or directed evolution [56–59]. Similarly, rAAV vectors that enter transduced cells are exposed to capsid degradation [60] and TLR9 activation [61,62]. Some groups have observed that mutating Tyr, Ser, and Thr residues on the AAV capsids limit capsid phosphorylation that leads to degradation and thus increases transduction efficiency [63–66]. TLR9 signaling leads to the production of interferon and proinflammatory cytokines, which can promote killing of transduced cells by cytotoxic T cells. Others have shown effective rAAV inoculations using a TLR9 antagonist to prevent an innate immune response [62].

The expressed transgene is also an immune target. Indeed, we showed that two of four Rh macaques developed antibodies against rh-eCD4-Ig [43^▪▪^]. However, those two macaques were able to maintain rh-eCD4-Ig levels high enough to protect from SHIV challenges. In contrast, in our own studies, four bNAbs bearing rhesus macaque constant regions elicited very high levels of anti-bNAb antibodies that abrogated most expression and protection. Other groups have examined rAAV-delivered SIV and HIV-1 antibodies in macaques, again without as much success. Fuchs *et al.* [67] showed that expressed SIV antibodies 4L6 and 5L7 were targeted by antibodies in macaques that limited their efficacy against SIV challenges. In a recent study by Martinez-Navio *et al.* [68^▪▪^], eight of eight macaques that expressed rAAV-delivered rhesusized bNAbs 3BNC117, 10-1074, 10E8, 1NC9, and 8ANC195 developed anti-transgene antibodies that likely contributed to their low expression levels and lack of therapeutic efficacy. The severity of antitransgene response directly correlated to the amount of somatic hypermutation of each antibody. In a similar study, Saunders *et al.* [69] were able to overcome antibody responses to their rAAV-delivered simianized bNAbs by treating the Rh macaques with cyclosporin A. Mouse studies have examined microRNA binding sites in the gene cassette that limit antigen-presenting cell transduction [70], administration of CTLA4-Ig at the time of inoculation [71], or cotransduction of PD-L1 to limit host responses to immunogenic transgenes [71]. Another option would be to use rAAV8 vectors that target the liver as well as muscle tissue [72]. Liver transduction establishes tolerance to the expressed transgene and can be followed with a different AAV serotype for an additional boost. Studies of AAV-expressed antibodies demonstrate that expressed transgenes that are more unlike self are more likely to elicit an immune response [43^▪▪^,^67^,^68^^▪▪^,^69^,^73^]. Here again, eCD4-Ig has an advantage in that it is more self-like than most bNAbs. Its only nonself regions are at the junctions of its three domains and the CCR5-mimetic peptide. The self-like quality of eCD4-Ig likely explains why antitransgene responses are modest and usually disappear over time.

A final hurdle to the use of rAAV as a vaccine alternative is the development of an effective kill switch. Although some high-risk or noncompliant individuals may benefit from AAV-eCD4-Ig, or AAV-expressed antibodies, the risk of a rare adverse event precludes wider use of this approach. Moreover, FDA (Food and Drug Administration) approval likely hinges on the existence of a kill switch that may even be critical to early human trials of an AAV-based vaccine. Intensive efforts to develop ways to permanently inactivate an AAV transgene are underway, and the problem appears considerably more tractable than the problem of developing a conventional vaccine. Moreover, these efforts can facilitate other uses of rAAV, for example, expressing antibodies that protect from diseases like malaria, or delivering biologics that are now being regularly injected.

## CONCLUSION

The discovery and characterization of bNAbs over the last decade has generated numerous inhibitors that can potently neutralize many HIV-1 isolates. These inhibitors have significant advantages over single antibody therapy in that they can simultaneously target more than one epitope on Env, localize the inhibitor to the site of entry, or limit pathways of escape by binding conserved sites on Env. rAAV vectors appear to be a viable way to express some of these inhibitors, and these vectors can provide long-term protection from HIV-1. However, more research on rAAV gene delivery is needed to overcome its current limitations, and a kill switch would likely be required if this approach is to be used broadly. Improving these engineered inhibitors and overcoming the limitation of rAAV should be high-priority goals because this approach can demonstrably provide sustained, effective, and universal protection from HIV-1.

## Acknowledgements

*The authors would like to thank Meredith Gardner, Ina Fetzer, and Christoph Fellinger for their comments and edits of the article. The authors would also like to thank Meredith Gardner for her help with the figures.*

### Financial support and sponsorship

*M.R.G. is supported by the NIAID Ruth L. Kirschstein NRSA F32 AI122980. M.F. is supported by the National Institutes of Health grants P01 AI100263, R37 AI091476, and by the Bill and Melinda Gates Foundation OPP1132169.*

### Conflicts of interest

*M.R.G. and M.F. both are cofounders and part-owners of Emmune, Inc. and own patents based on eCD4-Ig-related technologies.*

## REFERENCES AND RECOMMENDED READING

Papers of particular interest, published within the annual period of review, have been highlighted as:

▪ of special interest
▪▪ of outstanding interest

## References

[1] Wilen CB Tilton JC Doms RW HIV: cell binding and entry. Cold Spring Harb Perspect Med 2012; 2:a006866. 22908191 10.1101/cshperspect.a006866 PMC3405824

[2] Burton DR Mascola JR Antibody responses to envelope glycoproteins in HIV-1 infection. Nat Immunol 2015; 16:571–576. 25988889 10.1038/ni.3158 PMC4834917

[3▪] Liu J Ghneim K Sok D Antibody-mediated protection against SHIV challenge includes systemic clearance of distal virus. Science 2016; 353:1045–1049. 27540005 10.1126/science.aag0491 PMC5237379

[4▪▪] Gautam R Nishimura Y Pegu A A single injection of anti-HIV-1 antibodies protects against repeated SHIV challenges. Nature 2016; 533:105–109. 27120156 10.1038/nature17677 PMC5127204

[5] Ko S-Y Pegu A Rudicell RS Enhanced neonatal Fc receptor function improves protection against primate SHIV infection. Nature 2014; 514:642–645. 25119033 10.1038/nature13612 PMC4433741

[6] Shingai M Nishimura Y Klein F Antibody-mediated immunotherapy of macaques chronically infected with SHIV suppresses viraemia. Nature 2013; 503:277–280. 24172896 10.1038/nature12746 PMC4133787

[7] Barouch DH Whitney JB Moldt B Therapeutic efficacy of potent neutralizing HIV-1-specific monoclonal antibodies in SHIV-infected rhesus monkeys. Nature 2013; 503:224–228. 24172905 10.1038/nature12744 PMC4017780

[8] Caskey M Schoofs T Gruell H Antibody 10–1074 suppresses viremia in HIV-1-infected individuals. Nat Med 2017; [Epub ahead of print]. 10.1038/nm.4268 PMC5467219 28092665

[9▪] Scheid JF Horwitz JA Bar-On Y HIV-1 antibody 3BNC117 suppresses viral rebound in humans during treatment interruption. Nature 2016; 535:556–560. 27338952 10.1038/nature18929 PMC5034582

[10▪] Bar KJ Sneller MC Harrison LJ Effect of HIV antibody VRC01 on viral rebound after treatment interruption. N Engl J Med 2016; 375:2037–2050. 27959728 10.1056/NEJMoa1608243 PMC5292134

[11] Lynch RM Boritz E Coates EE Virologic effects of broadly neutralizing antibody VRC01 administration during chronic HIV-1 infection. Sci Transl Med 2015; 7:319ra206–1319ra. 10.1126/scitranslmed.aad5752 PMC12366723 26702094

[12] Ledgerwood JE Coates EE Yamshchikov G Safety, pharmacokinetics and neutralization of the broadly neutralizing HIV-1 human monoclonal antibody VRC01 in healthy adults. Clin Exp Immunol 2015; 182:289–301. 26332605 10.1111/cei.12692 PMC4636891

[13] Caskey M Klein F Lorenzi JCC Viraemia suppressed in HIV-1-infected humans by broadly neutralizing antibody 3BNC117. Nature 2015; 522:487–491. 25855300 10.1038/nature14411 PMC4890714

[14▪▪] Huang J Kang Byong H Ishida E Identification of a CD4-binding-site antibody to HIV that evolved near-pan neutralization breadth. Immunity 2016; 45:1108–1121. 27851912 10.1016/j.immuni.2016.10.027 PMC5770152

[15] Huang J Ofek G Laub L Broad and potent neutralization of HIV-1 by a gp41-specific human antibody. Nature 2012; 491:406–412. 23151583 10.1038/nature11544 PMC4854285

[16] Wagh K Bhattacharya T Williamson C Optimal combinations of broadly neutralizing antibodies for prevention and treatment of HIV-1 clade C infection. PLoS Pathog 2016; 12:e1005520. 27028935 10.1371/journal.ppat.1005520 PMC4814126

[17▪] Gardner MR Fellinger CH Prasad NR CD4-induced antibodies promote association of the HIV-1 envelope glycoprotein with CD4-binding site antibodies. J Virol 2016; 90:7822–7832. 27334589 10.1128/JVI.00803-16 PMC4988152

[18] Klein F Halper-Stromberg A Horwitz JA HIV therapy by a combination of broadly neutralizing antibodies in humanized mice. Nature 2012; 492:118–122. 23103874 10.1038/nature11604 PMC3809838

[19] Doria-Rose NA Louder MK Yang Z HIV-1 neutralization coverage is improved by combining monoclonal antibodies that target independent epitopes. J Virol 2012; 86:3393–3397. 22258252 10.1128/JVI.06745-11 PMC3302320

[20] Zwick MB Wang M Poignard P Neutralization synergy of human immunodeficiency virus type 1 primary isolates by cocktails of broadly neutralizing antibodies. J Virol 2001; 75:12198–12208. 11711611 10.1128/JVI.75.24.12198-12208.2001 PMC116117

[21] Asokan M Rudicell RS Louder M Bispecific antibodies targeting different epitopes on the HIV-1 envelope exhibit broad and potent neutralization. J Virol 2015; 89:12501–12512. 26446600 10.1128/JVI.02097-15 PMC4665248

[22▪] Bournazos S Gazumyan A Seaman Michael S Bispecific anti-HIV-1 antibodies with enhanced breadth and potency. Cell 2016; 165:1609–1620. 27315478 10.1016/j.cell.2016.04.050 PMC4970321

[23] Galimidi RP Klein Joshua S Politzer Maria S Intra-spike crosslinking overcomes antibody evasion by HIV-1. Cell 2015; 160:433–446. 25635457 10.1016/j.cell.2015.01.016 PMC4401576

[24] Pace CS Song R Ochsenbauer C Bispecific antibodies directed to CD4 domain 2 and HIV envelope exhibit exceptional breadth and picomolar potency against HIV-1. Proc Natl Acad Sci U S A 2013; 110:13540–13545. 23878231 10.1073/pnas.1304985110 PMC3746901

[25▪▪] Huang Y Yu J Lanzi A Engineered bispecific antibodies with exquisite HIV-1-neutralizing activity. Cell 2016; 165:1621–1631. 27315479 10.1016/j.cell.2016.05.024 PMC4972332

[26] Wyatt R Sodroski J The HIV-1 envelope glycoproteins: fusogens, antigens, and immunogens. Science 1998; 280:1884–1888. 9632381 10.1126/science.280.5371.1884

[27] Rizzuto CD Wyatt R Hernández-Ramos N A conserved HIV gp120 glycoprotein structure involved in chemokine receptor binding. Science 1998; 280:1949–1953. 9632396 10.1126/science.280.5371.1949

[28] Kwong PD Wyatt R Robinson J Structure of an HIV gp120 envelope glycoprotein in complex with the CD4 receptor and a neutralizing human antibody. Nature 1998; 393:648–659. 9641677 10.1038/31405 PMC5629912

[29] Olshevsky U Helseth E Furman C Identification of individual human immunodeficiency virus type 1 gp120 amino acids important for CD4 receptor binding. J Virol 1990; 64:5701–5707. 2243375 10.1128/jvi.64.12.5701-5707.1990 PMC248709

[30] Cordonnier A Montagnier L Emerman M Single amino-acid changes in HIV envelope affect viral tropism and receptor binding. Nature 1989; 340:571–574. 2475780 10.1038/340571a0

[31] Kowalski M Potz J Basiripour L Functional regions of the envelope glycoprotein of human immunodeficiency virus type 1. Science 1987; 237:1351–1355. 3629244 10.1126/science.3629244

[32] Chen W Feng Y Gong R Engineered single human CD4 domains as potent HIV-1 inhibitors and components of vaccine immunogens. J Virol 2011; 85:9395–9405. 21715496 10.1128/JVI.05119-11 PMC3165761

[33] Quinlan BD Gardner MR Joshi VR Direct expression and validation of phage-selected peptide variants in mammalian cells. J Biol Chem 2013; 288:18803–18810. 23667257 10.1074/jbc.M113.452839 PMC3696656

[34] Thali M Moore JP Furman C Characterization of conserved human immunodeficiency virus type 1 gp120 neutralization epitopes exposed upon gp120-CD4 binding. J Virol 1993; 67:3978–3988. 7685405 10.1128/jvi.67.7.3978-3988.1993 PMC237765

[35] Xiang S-H Wang L Abreu M Epitope mapping and characterization of a novel CD4-induced human monoclonal antibody capable of neutralizing primary HIV-1 strains. Virology 2003; 315:124–134. 14592765 10.1016/s0042-6822(03)00521-x

[36] Choe H Li W Wright PL Tyrosine sulfation of human antibodies contributes to recognition of the CCR5 binding region of HIV-1 gp120. Cell 2003; 114:161–170. 12887918 10.1016/s0092-8674(03)00508-7

[37] Chen W Zhu Z Feng Y Dimitrov DS Human domain antibodies to conserved sterically restricted regions on gp120 as exceptionally potent cross-reactive HIV-1 neutralizers. Proc Natl Acad Sci U S A 2008; 105:17121–17126. 18957538 10.1073/pnas.0805297105 PMC2579388

[38] Lagenaur LA Villarroel VA Bundoc V sCD4-17b bifunctional protein: Extremely broad and potent neutralization of HIV-1 Env pseudotyped viruses from genetically diverse primary isolates. Retrovirology 2010; 7:11. 20158904 10.1186/1742-4690-7-11 PMC2843639

[39] West AP Galimidi RP Foglesong CP Evaluation of CD4–CD4i antibody architectures yields potent, broadly cross-reactive anti-human immunodeficiency virus reagents. J Virol 2010; 84:261–269. 19864392 10.1128/JVI.01528-09 PMC2798438

[40] Quinlan BD Joshi VR Gardner MR A double-mimetic peptide efficiently neutralizes HIV-1 by bridging the CD4- and coreceptor-binding sites of gp120. J Virol 2014; 88:3353–3358. 24390333 10.1128/JVI.03800-13 PMC3957931

[41] Chen W Feng Y Prabakaran P Exceptionally potent and broadly cross-reactive, bispecific multivalent HIV-1 inhibitors based on single human CD4 and antibody domains. J Virol 2014; 88:1125–1139. 24198429 10.1128/JVI.02566-13 PMC3911630

[42] Sun M Pace CS Yao X Rational design and characterization of the novel, broad and potent bispecific HIV-1 neutralizing antibody iMabm36. J Acquir Immune Defic Syndr 2014; 66:473–483. 24853313 10.1097/QAI.0000000000000218 PMC4163016

[43▪▪] Gardner MR Kattenhorn LM Kondur HR AAV-expressed eCD4-Ig provides durable protection from multiple SHIV challenges. Nature 2015; 519:87–91. 25707797 10.1038/nature14264 PMC4352131

[44] Chiang JJ Gardner MR Quinlan BD Enhanced recognition and neutralization of HIV-1 by antibody-derived CCR5-mimetic peptide variants. J Virol 2012; 86:12417–12421. 22933279 10.1128/JVI.00967-12 PMC3486504

[45] Dorfman T Moore MJ Guth AC A tyrosine-sulfated peptide derived from the heavy-chain CDR3 region of an HIV-1-neutralizing antibody binds gp120 and inhibits HIV-1 infection. J Biol Chem 2006; 281:28529–28535. 16849323 10.1074/jbc.M602732200

[46] Nathwani AC Reiss UM Tuddenham EGD Long-term safety and efficacy of factor IX gene therapy in hemophilia B. N Engl J Med 2014; 371:1994–2004. 25409372 10.1056/NEJMoa1407309 PMC4278802

[47] Jiang H Pierce GF Ozelo MC Evidence of multiyear factor IX expression by AAV-mediated gene transfer to skeletal muscle in an individual with severe hemophilia B. Mol Ther 2006; 14:452–455. 16822719 10.1016/j.ymthe.2006.05.004

[48] Fang J Qian J-J Yi S Stable antibody expression at therapeutic levels using the 2A peptide. Nat Biotechnol 2005; 23:584–590. 15834403 10.1038/nbt1087

[49] Lewis AD Chen R Montefiori DC Generation of neutralizing activity against human immunodeficiency virus type 1 in serum by antibody gene transfer. J Virol 2002; 76:8769–8775. 12163597 10.1128/JVI.76.17.8769-8775.2002 PMC136414

[50] Fuchs SP Martinez-Navio JM Gao G Desrosiers RC Recombinant AAV vectors for enhanced expression of authentic IgG. PLoS One 2016; 11:e0158009. 27332822 10.1371/journal.pone.0158009 PMC4917256

[51] Kotterman MA Yin L Strazzeri JM Antibody neutralization poses a barrier to intravitreal adeno-associated viral vector gene delivery to non-human primates. Gene Ther 2015; 22:116–126. 25503696 10.1038/gt.2014.115 PMC4393652

[52] Hareendran S Balakrishnan B Sen D Adeno-associated virus (AAV) vectors in gene therapy: immune challenges and strategies to circumvent them. Rev Med Virol 2013; 23:399–413. 24023004 10.1002/rmv.1762

[53] Rogers G Martino A Aslanidi G Innate immune responses to AAV vectors. Front Microbiol 2011; 2:194. 21954398 10.3389/fmicb.2011.00194 PMC3175613

[54] Calcedo R Wilson J Humoral immune response to AAV. Front Immunol 2013; 4:341. 24151496 10.3389/fimmu.2013.00341 PMC3799231

[55] Boutin S Monteilhet V Veron P Prevalence of serum IgG and neutralizing factors against adeno-associated virus (AAV) types 1, 2, 5, 6, 8, and 9 in the healthy population: implications for gene therapy using AAV vectors. Hum Gene Ther 2010; 21:704–712. 20095819 10.1089/hum.2009.182

[56] Zinn E Pacouret S Khaychuk V In silico reconstruction of the viral evolutionary lineage yields a potent gene therapy vector. Cell Rep 2015; 12:1056–1068. 26235624 10.1016/j.celrep.2015.07.019 PMC4536165

[57] Davis AS Federici T Ray WC Rational design and engineering of a modified adeno-associated virus (AAV1)-based vector system for enhanced retrograde gene delivery. Neurosurgery 2015; 76:216–225. 25549186 10.1227/NEU.0000000000000589

[58] Lochrie MA Tatsuno GP Christie B Mutations on the external surfaces of adeno-associated virus type 2 capsids that affect transduction and neutralization. J Virol 2006; 80:821–834. 16378984 10.1128/JVI.80.2.821-834.2006 PMC1346838

[59] Mao Y Wang X Yan R Single point mutation in adeno-associated viral vectors-DJ capsid leads to improvement for gene delivery in vivo. BMC Biotechnol 2016; 16:1. 26729248 10.1186/s12896-015-0230-0 PMC4700607

[60] Zhong L Li B Jayandharan G Tyrosine-phosphorylation of AAV2 vectors and its consequences on viral intracellular trafficking and transgene expression. Virology 2008; 381:194–202. 18834608 10.1016/j.virol.2008.08.027 PMC2643069

[61] Zhu J Huang X Yang Y The TLR9-MyD88 pathway is critical for adaptive immune responses to adeno-associated virus gene therapy vectors in mice. J Clin Invest 2009; 119:2388–2398. 19587448 10.1172/JCI37607 PMC2719948

[62] Martino AT Suzuki M Markusic DM The genome of self-complementary adeno-associated viral vectors increases Toll-like receptor 9-dependent innate immune responses in the liver. Blood 2011; 117:6459–6468. 21474674 10.1182/blood-2010-10-314518 PMC3123017

[63] Zhong L Li B Mah CS Next generation of adeno-associated virus 2 vectors: point mutations in tyrosines lead to high-efficiency transduction at lower doses. Proc Natl Acad Sci U S A 2008; 105:7827–7832. 18511559 10.1073/pnas.0802866105 PMC2402387

[64] Martino AT Basner-Tschakarjan E Markusic DM Engineered AAV vector minimizes in vivo targeting of transduced hepatocytes by capsid-specific CD8^+^ T cells. Blood 2013; 121:2224–2233. 23325831 10.1182/blood-2012-10-460733 PMC3606062

[65] Gabriel N Hareendran S Sen D Bioengineering of AAV2 capsid at specific serine, threonine, or lysine residues improves its transduction efficiency in vitro and in vivo. Hum Gene Ther Methods 2013; 24:80–93. 23379478 10.1089/hgtb.2012.194 PMC3732126

[66] Aslanidi GV Rivers AE Ortiz L Optimization of the capsid of recombinant adeno-associated virus 2 (AAV2) vectors: the final threshold? PLoS One 2013; 8:e59142. 23527116 10.1371/journal.pone.0059142 PMC3602601

[67] Fuchs SP Martinez-Navio JM Piatak M Jr AAV-delivered antibody mediates significant protective effects against SIVmac239 challenge in the absence of neutralizing activity. PLoS Pathog 2015; 11:e1005090. 26248318 10.1371/journal.ppat.1005090 PMC4527674

[68▪▪] Martinez-Navio JM Fuchs SP Pedreño-López S Host antiantibody responses following adeno-associated virus-mediated delivery of antibodies against HIV and SIV in Rhesus Monkeys. Mol Ther 2016; 24:76–86. 26444083 10.1038/mt.2015.191 PMC4754551

[69] Saunders KO Pegu A Georgiev IS Sustained delivery of a broadly neutralizing antibody in nonhuman primates confers long-term protection against simian/human immunodeficiency virus infection. J Virol 2015; 89:5895–5903. 25787288 10.1128/JVI.00210-15 PMC4442454

[70] Majowicz A Maczuga P Kwikkers KL Mir-142-3p target sequences reduce transgene-directed immunogenicity following intramuscular adeno-associated virus 1 vector-mediated gene delivery. J Gene Med 2013; 15:219–232. 23658149 10.1002/jgm.2712

[71] Adriouch S Franck E Drouot L Improved immunological tolerance following combination therapy with CTLA-4/Ig and AAV-mediated PD-L1/2 muscle gene transfer. Front Microbiol 2011; 2:199. 22046170 10.3389/fmicb.2011.00199 PMC3202221

[72] Lu Y Song S Distinct immune responses to transgene products from rAAV1 and rAAV8 vectors. Proc Natl Acad Sci U S A 2009; 106:17158–17162. 19805176 10.1073/pnas.0909520106 PMC2761323

[73] Johnson PR Schnepp BC Zhang J Vector-mediated gene transfer engenders long-lived neutralizing activity and protection against SIV infection in monkeys. Nat Med 2009; 15:901–906. 19448633 10.1038/nm.1967 PMC2723177

